# Free exopolysaccharide from *Mycoplasma mycoides* subsp. *mycoides* possesses anti-inflammatory properties

**DOI:** 10.1186/s13567-015-0252-6

**Published:** 2015-10-21

**Authors:** Philippe Totté, Carinne Puech, Valérie Rodrigues, Clothilde Bertin, Lucia Manso-Silvan, François Thiaucourt

**Affiliations:** Centre International de Recherche en Agronomie pour le Développement, UMR CMAEE, Montpellier, France; Institut National de Recherche Agronomique, UMR1309 CMAEE, Montpellier, France

## Abstract

In this study we explored the immunomodulatory properties of highly purified free galactan, the soluble exopolysaccharide secreted by *Mycoplasma mycoides* subsp. *mycoides* (*Mmm*). Galactan was shown to bind to TLR2 but not TLR4 using HEK293 reporter cells and to induce the production of the anti-inflammatory cytokine IL-10 in bovine macrophages, whereas low IL-12p40 and no TNF-α, both pro-inflammatory cytokines, were induced in these cells. In addition, pre-treatment of macrophages with galactan substantially reduced lipopolysaccharide (LPS)-induced production of pro-inflammatory cytokines TNF- and IL-12p40 while increasing LPS-induced secretion of immunosuppressive IL-10. Also, galactan did not activate naïve lymphocytes and induced only low production of the Th1 cytokine IFN-γ in *Mmm*-experienced lymphocytes. Finally, galactan triggered weak recall proliferation of CD4+ T lymphocytes from contagious bovine pleuropneumonia-infected animals despite having a positive effect on the expression of co-stimulatory molecules on macrophages. All together, these results suggest that galactan possesses anti-inflammatory properties and potentially provides *Mmm* with a mechanism to evade host innate and adaptive cell-mediated immune responses.

**Electronic supplementary material:**

The online version of this article (doi:10.1186/s13567-015-0252-6) contains supplementary material, which is available to authorized users.

## Introduction

Contagious bovine pleuropneumonia (CBPP) is a contagious respiratory disease caused by *Mycoplasma mycoides* subsp*. mycoides* small colony biotype (*Mmm*) and notifiable to the World Organization for Animal Health [1]. Respiratory distress occurs in acute forms of CBPP frequently followed by death in the absence of antibiotic treatment. Lesions in the thoracic cavity are suggestive of an overwhelming inflammatory response developed by the host. The prominent lesions in the lung include: thrombosis of lymphatics and blood vessels; interlobular edema; hepatisation and consolidation of individual lobules leading to a marbled appearance of the lung, and yellow or turbid exudates in the pleural cavity (reviewed in [2]). In addition, archetypal inflammatory cytokines are detected in vitro in supernatants of macrophages stimulated with several *Mmm* strains and in vivo in the plasma of cattle during the course of experimental infection with virulent *Mmm* [3, 4].

The mechanisms and components of *Mmm* involved in immunomodulation in general and in host inflammatory responses in particular are poorly characterized. Available *Mmm* sequenced genomes [5, 6] indicate that classical virulence determinants such as toxins, invasins or cytolysins, are absent in *Mmm*. More probably, the virulence factors of mycoplasma species seem to be determined by intrinsic metabolic pathway functions [7, 8] or by constituents of the mycoplasmal outer surface including lipoproteins and polysaccharides. *Mmm* cells secrete galactan, a homopolysaccharide consisting of β1 → 6 galactofuranosyl sub-units and present as a capsular form (CPS) and as a free extracellular form hereafter termed exopolysaccharide (EPS) [9, 10]. While CPS and EPS possess the same type of homopolysaccharide, the former differs from the latter as it is linked to a lipid anchored moiety, probably a diglycosyldiacylglycerol residue as for *Mycoplasma genitalium* [11]. The physical and biological properties of CPS and EPS may therefore vary considerably. Galactan EPS is secreted in high quantities by *Mmm* that multiply in the lung lesions and, because it is soluble, it is found in large amounts in the blood of infected cattle [12]. Biological effects of crude galactan observed after intravenous injection in cattle include: increased pulmonary arterial and decreased systemic arterial blood pressures, hemorrhages associated with alveolar ducts and vessel walls, areas of pulmonary edema, induced lesions in joints and kidneys and a prolonged mycoplasmaemia after subcutaneous injection of live *Mmm* [13, 14]. These results suggest a possible role for galactan as a pro-inflammatory factor although the contribution of contaminating components such as polypeptides and non-*Mmm* polysaccharides present in the enriched culture medium cannot be excluded. Recently, we reported a method using a chemically defined synthetic medium to maintain *Mmm* viability and allow the preparation of free galactan EPS of high purity as shown by silver staining on SDS-PAGE and nuclear magnetic resonance spectroscopy [10].

Macrophages express a large repertoire of pathogen-recognition receptors, including toll-like receptors (TLRs), and are key players in the initiation of inflammatory responses during infection [15]. Inflammation is a highly complex response under the control of a network of cytokines with either pro-inflammatory effects such as IL-1β, IL-12p40, TNF-α, IFN-γ, or anti-inflammatory properties such as IL-10 and TGF-β (reviewed in [16]). Bacterial polysaccharides are capable of inducing the production of pro-inflammatory cytokines by macrophages through interaction with TLR2 and/or TLR4 [17–21]. In addition, lymphocytes of the Th1 subset may also trigger or exacerbate inflammation as a result of interactions with bacterial polysaccharides through a mechanism depending on both TLR2 and antigen presentation [18, 22]. Importantly, antigen presentation to lymphocytes by antigen presenting cells (APC) such as dendritic cells and macrophages requires expression of co-stimulatory molecules including CD40, CD80, and CD86 [23].

In an effort to address the pathogenesis of CBPP, we investigated the interactions of highly purified galactan EPS, the free exopolysaccharide of *Mmm*, with bovine cells involved in both innate and acquired immunity. First of all, binding of galactan to TLRs was analyzed using highly sensitive transformed cell lines. Then, the effects of galactan on cytokines production and expression of co-stimulatory molecules by bovine macrophages were characterized. Finally, the capacity of galactan to trigger a pro-inflammatory Th1-like response from lymphocytes of naïve and CBPP-infected animals was investigated.

## Materials and methods

### Ethics statement

The use of cattle as a source of macrophages and lymphocytes was approved by the Languedoc-Roussillon Animal Ethics Committee (CEEA-LR No. 36) and carried out in strict accordance with the French Ministry of Agriculture’s guidelines for use of animals for scientific purposes.

### Preparation of galactan and whole antigen from *Mmm*

*Mmm* strains Afadé and T1/44 were used for galactan purification and stimulation of lymphocytes respectively. For the preparation of highly pure galactan EPS, *Mmm* Afadé was first grown in PPLO-based medium before transfer in chemically defined CMRL-1066 medium (Invitrogen, France) in which de novo synthesis of polysaccharides was previously shown [10]. To purify the galactan EPS, cells were removed by centrifugation and polypeptides were precipitated from the supernatant with 1/10 volume of cold 100% (w/v) trichloroacetic acid (Sigma-Aldrich) at 4 °C for 2 h followed by centrifugation (14 000 *g*, 60 min, 4 °C). Polysaccharides contained in the supernatant were precipitated with 6–10 volumes of cold acetone at −20 °C for 48 h and collected after centrifugation (14 000 *g*, 60 min, 4 °C). The acetone was carefully removed and the pellet was air-dried and dissolved in ultra-pure sterile water and dialyzed in 3.5 kDa cut-off dialysis tubing (Spectrum Laboratories) to remove small peptides. Galactan concentration was estimated by the phenol/sulfuric acid method using glucose as standard and stored at 4 °C until used [10].

Killed *Mmm* were also prepared from log phase T1/44 cultures grown in PPLO and washed two times in PBS before protein titration by the bicinchoninic acid method. The T1/44 strain of *Mmm* was chosen as a positive control of antigen-specific proliferation as shown in previous work [24]. *Mmm* organisms had to be inactivated before use in co-cultures with lymphocytes to avoid direct killing of cells [25]. Whole inactivated *Mmm* antigen was obtained after heating at 60 °C for 1 h and was stored at −20 °C until used.

### Macrophages and cell lines expressing TLRs

For macrophages, heparinized blood was collected from the jugular vein of healthy cattle and peripheral blood mononuclear cells (PBMC) were purified using standard procedures. Monocytes were separated from other cells by positive selection of CD14+ cells using anti-human CD14 magnetic microbeads (Miltenyi Biotec GmbH, Germany) according to the manufacturer’s instructions and resuspended in Iscove’s modified Dulbecco’s medium (IMDM) supplemented with 2 mM l-glutamine, 50 μM 2-mercaptoethanol, 50 µg/mL gentamycin (Life Technologies, France) and 10% heat inactivated fetal calf serum (FCS; Eurobio AbCys, France). CD14+ cells were seeded at 1 × 10^6^ cells/mL in 24-well plates and incubated for 5 days at 37 °C and 5% CO_2_. For stimulation assays, macrophages were incubated for 48 h at 37 °C and 5% CO_2_ with different concentrations of galactan or 2 μg/mL of lipopolysaccharide (LPS) from *Escherichiacoli* 0111:B4 (Invivogen, France) as a positive control. Supernatants were centrifuged at 300 × *g* to remove cells before storage at −20 °C.

HEK-Blue™ mTLR2 (HEK-TLR2) and HEK-Blue™ mTLR4 (HEK-TLR4) cell lines (Invivogen, France) are derivative of HEK293 cells obtained respectively by co-transfection of the mouse TLR2 or TLR4 and secreted embryonic alkaline phosphatase (SEAP) genes. Ligand activation of TLR induces production of SEAP and QUANTI-Blue™ (Invivogen, France), a colorimetric enzyme assay, allows quantification of SEAP in supernatants using a spectrophotometer at 620-655 nm. HEK-Blue™ Null1 (HEK-Null1) and HEK-Blue™ Null2 (HEK-Null2) cell lines (Invivogen, France) are parental cell lines transfected only with the SEAP gene. For stimulation assays, cells were seeded in 96-wells plates in recommended media (Invivogen, France) and incubated for 20 h at 37 °C and 5% CO_2_ with different concentrations of EPS, and with specific TLR agonists (Invivogen, France): 100 ng/mL of synthetic diacylated lipoprotein (FSL1) for TLR2, and 10 ng/mL of LPS from *E. coli* 0111:B4 for TLR4. Supernatants were titrated for alkaline phosphatase activity in duplicates.

### Lymphoproliferative assays

Cells were prepared from mediastinal lymph nodes draining the lungs of naïve and CBPP-infected cattle (also called *Mmm*-experienced cells) and stored in liquid nitrogen as previously described [26]. Upon thawing, cells were loaded with carboxyfluorescein diacetate succinimidyl ester (CFSE, Invitrogen, France) at a final concentration of 1 μM before incubation for 7 days with 5 μg/mL of heat-inactivated *Mmm* or 2.5 μg/mL of the mitogen Concanavalin A (ConA) as a positive control. Cells from CBPP-infected and naïve animals showed similar reactivity to ConA and only assays in which ConA controls were within an acceptable range were analyzed further. Galactan was used at a final concentration of 10 μg/mL unless specified otherwise. Finally, purified protein derivative from *Mycobacterium avium* subsp. *paratuberculosis* (PPDa) cultures (Prionics AG, Switzerland) was used as irrelevant antigen to measure non-specific proliferation.

### Flow cytometry

Lymph node cells were surface stained with the following primary monoclonal antibodies (mAb): mAb IL-A11 (IgG2a) for CD4 (VMRD, USA). Macrophages were stained with mAb IL-A156 for CD40, and mAb IL-A159 for CD80 (all IgG1 isotype from AbD Serotec, UK). Cells were then washed and stained with fluorochrome-conjugated, isotype-specific antibodies. Debris and dead cells were excluded from analysis based on granularity and size and, for lymphocytes, based on inclusion of 7-amino-actinomycin D (7-AAD) as a viability dye (BD Biosciences, USA). Multiple color analyses were performed with a flow cytometer (FACScanto; Becton–Dickinson) equipped with the FACSDiva software (BD Biosciences) after the acquisition of at least 5000 events. Control isotype antibodies (AbD Serotec, UK) were used to evaluate nonspecific binding and to set gates and quadrants delineating positive populations. Results were expressed as percentages of positively labeled cells and/or geometric mean fluorescence intensity (MFI). Proliferation levels were expressed as percentages of CFSE^low^ cells which correspond to decreased CFSE fluorescence intensity relative to that in undivided cells.

### Cytokines ELISA

For bovine IFN-γ, the Bovigam™ ELISA kit (Prionics AG, Switzerland) was used according to the manufacturer’s instructions and results are expressed as mean optical densities of duplicate assays. For bovine IL-10, IL-12p40, and TNF-α, matched antibody pairs (AbD Serotec, UK) were used in sandwich ELISA as described previously [27] with minor modifications. Briefly, 96 wells NUNC MaxiSorp™ plates (Thermo Fisher Scientific, Germany) were coated with capture antibodies and washed after blocking. After incubating the plates with culture supernatants in duplicates, and washing, specific detection antibodies coupled to biotin were added. Serial dilutions of recombinant bovine cytokines were used to generate a calibration curve. After washing, the plates were incubated with streptavidin coupled to horseradish peroxidase (Sigma-Aldrich, France) and washed before adding a solution containing tetramethylbenzidine (TMB). The reaction was blocked with H_2_SO_4_ at 1 M and plates were read at 450 nm using an ELISA reader. Results are expressed as mean protein concentrations.

### Statistics

A nonparametric Mann–Whitney U-test [28] was used to analyze differences between responses obtained with various stimuli. A difference was considered to be significant at a *p* value of <0.05.

## Results

### Galactan is recognized by TLR2 but not TLR4

**Figure 1:**
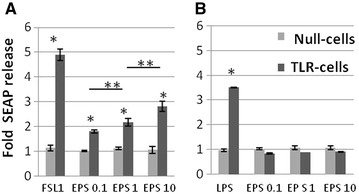
**Effect of free** ***Mmm*** **galactan on secreted embryonic alkaline phosphatase (SEAP) release from HEK-Blue mTLR2 cells (** **A** **) and HEK-Blue mTLR4 cells (** **B** **)**. Null-cells (i.e., HEK-Blue cells neither expressing TLR2 nor TLR4) or TLR-cells were incubated with galactan (EPS) at 0.1, 1 and 10 µg/mL, or with FSL1 lipopeptide and *E. coli* LPS as positive controls for TLR2 and TLR4 cells respectively. SEAP release after stimulation was measured by a colorimetric method and expressed as mean fold changes (±SD) in comparison to unstimulated cells (*n* = 3). *p < 0.001 relative to unstimulated controls; **p < 0.05 between different EPS concentrations.

The level of SEAP protein released into the culture media by TLR-transfected HEK cells was used to quantify the extent of TLR2 or TLR4 stimulation which also correlates with the level of NF-κB activation. As shown in Figure 1, galactan triggered a significant release of SEAP protein by HEK cells expressing TLR2. The activity of galactan was detected even at the lowest concentration and was dose dependent. In contrast, no activity of galactan was detected with null-cells or with cells expressing TLR4.

### Galactan induces an anti-inflammatory cytokine response in bovine macrophages and inhibits the pro-inflammatory activity of LPS

**Figure 2:**
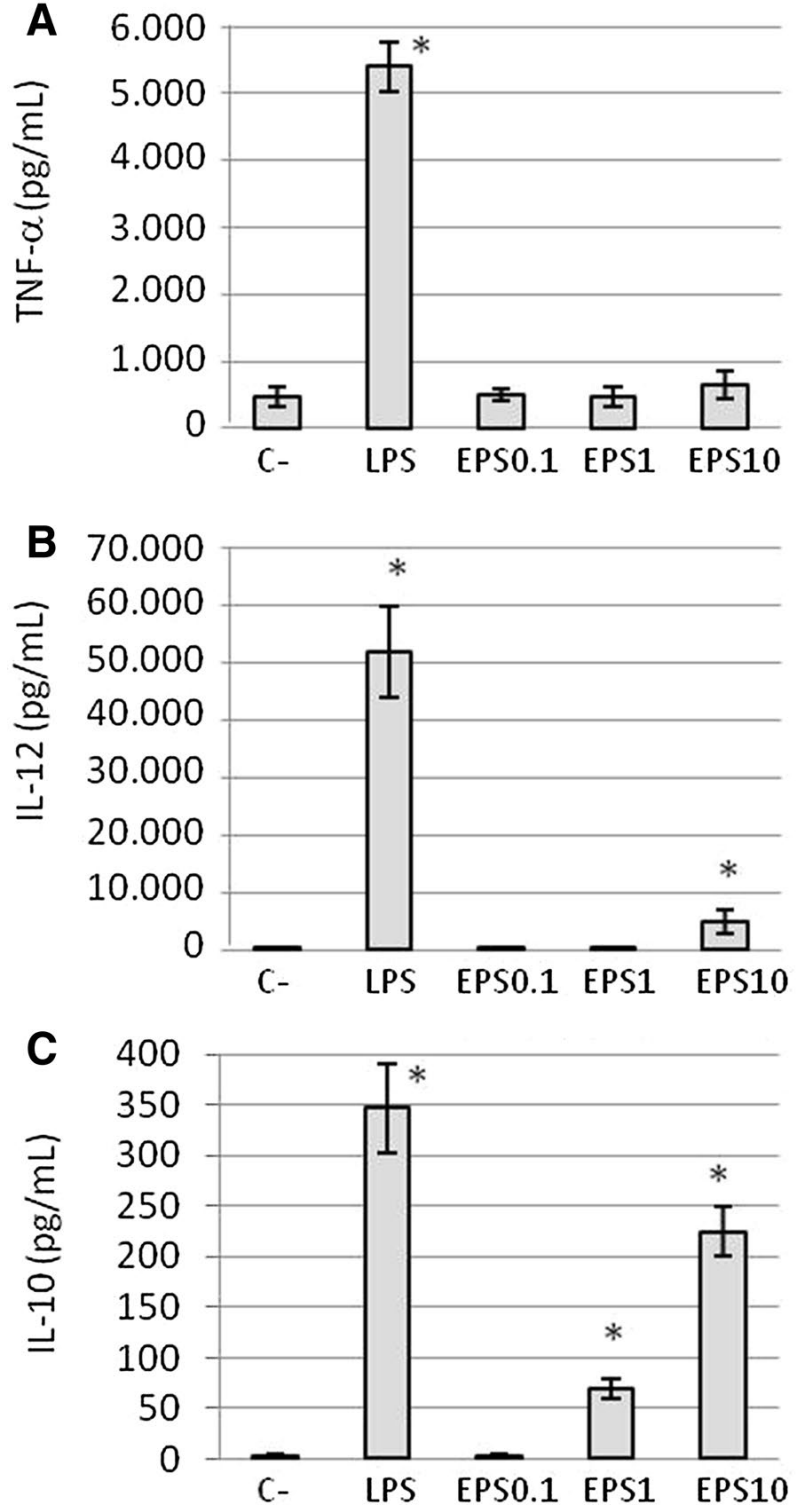
**Effect of free** ***Mmm*** **galactan on TNF-α (** **A** **), IL-12p40 (** **B** **), and IL-10 (** **C** **) release by bovine macrophages.** Cells were incubated for 48 h with galactan (EPS) at 0.1, 1 and 10 µg/mL, or with *E. coli* LPS and medium only as positive and negative (C−) controls respectively. Estimation of cytokine production was performed by ELISA. Results are expressed as mean (±SD) protein concentrations of three different experiments. Asterisks indicate statistically significant differences (*p* < 0.05) with the negative control.

As shown in Figure 2, TNF-α was not produced by macrophages in response to galactan and low levels of IL-12p40 were detected but only at the highest concentration used. This was not due to an intrinsic lack of response by macrophages as shown by a substantial production in the presence of LPS. In contrast, a significant induction of IL-10 was detected after stimulation with 1 μg/mL of galactan. The production of IL-10 in response to galactan was dose-dependent as 10 µg/mL induced a substantially higher response.

**Figure 3:**
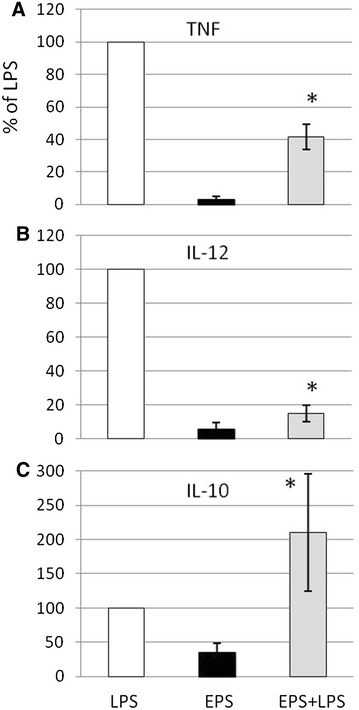
**Effect of pre-treatment with free** ***Mmm*** **galactan on LPS-induced release of TNF-α (** **A** **), IL-12p40 (** **B** **), and IL-10 (** **C** **) by bovine macrophages.** Cells were incubated with LPS alone at 2 µg/mL (LPS), galactan alone at 10 µg/mL (EPS), or galactan for 8 h before stimulation with 2 µg/mL of LPS (EPS + LPS). ELISA was used to detect levels of secreted cytokines in 2-day-old supernatants. Results from three different experiments are expressed as mean (±SD) percentage of the value obtained with LPS alone. Asterisks indicate statistically significant differences (*p* < 0.05) between LPS alone and EPS + LPS.

When macrophages were treated with galactan prior to LPS stimulation, TNF-α and IL-12p40 responses triggered by LPS were reduced by 55 ± 9 and 88 ± 3% respectively (Figure 3). On the other hand, the production of IL-10 significantly increased although with a high amplitude in the effect of galactan between different experiments (i.e., from 130 to 300% as shown in Figure 3).

### Cell-surface expression of co-stimulatory molecules on bovine macrophages is increased by galactan

**Figure 4:**
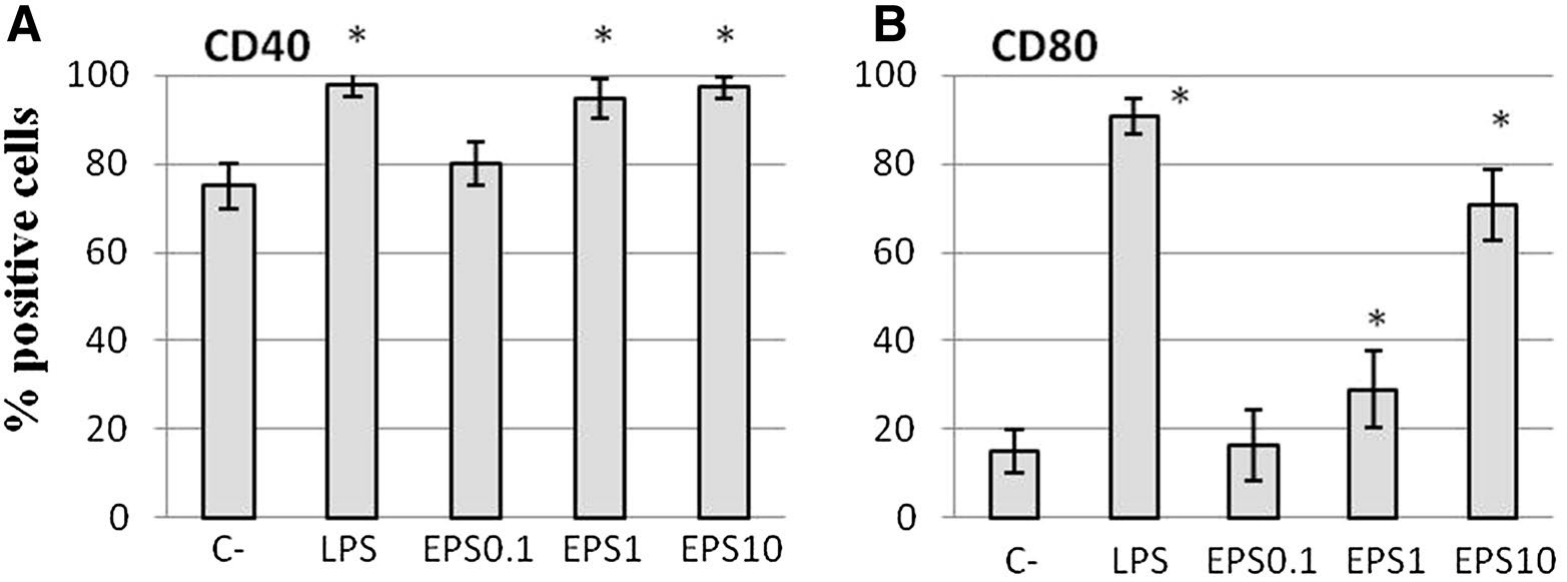
**Effect of free** ***Mmm*** **galactan on surface expression of CD40 (** **A** **) and CD80 (** **B** **) on bovine macrophages.** Cells were incubated for 48 h with galactan (EPS) at 0.1, 1 and 10 µg/mL, or with LPS and medium only (C−) as positive and negative controls respectively. Cell-surface expression of CD40/CD80 was measured by flow cytometry and results expressed as mean (±SD) percentages of positive cells for a given marker (*n* = 3). Asterisks indicate statistically significant differences (*p* < 0.05) with the negative control.

Although around 80% of macrophages constitutively expressed CD40 (Figure 4A), the proportion of positive cells reached 100% of the total population in the presence of galactan. The positive effect of galactan on CD40 expression was confirmed by a substantial increase in the mean fluorescence intensity (MFI) which is an estimation of the mean number of CD40 molecules per cell (Additional file 1). In addition, cell surface expression of CD80 was strongly induced by galactan with up to a fourfold increase observed at the highest dose (Figure 4B).

### CD4+ T cells from CBPP-infected cattle but not naïve animals proliferate in response to galactan and produce low levels of IFN-γ

**Figure 5:**
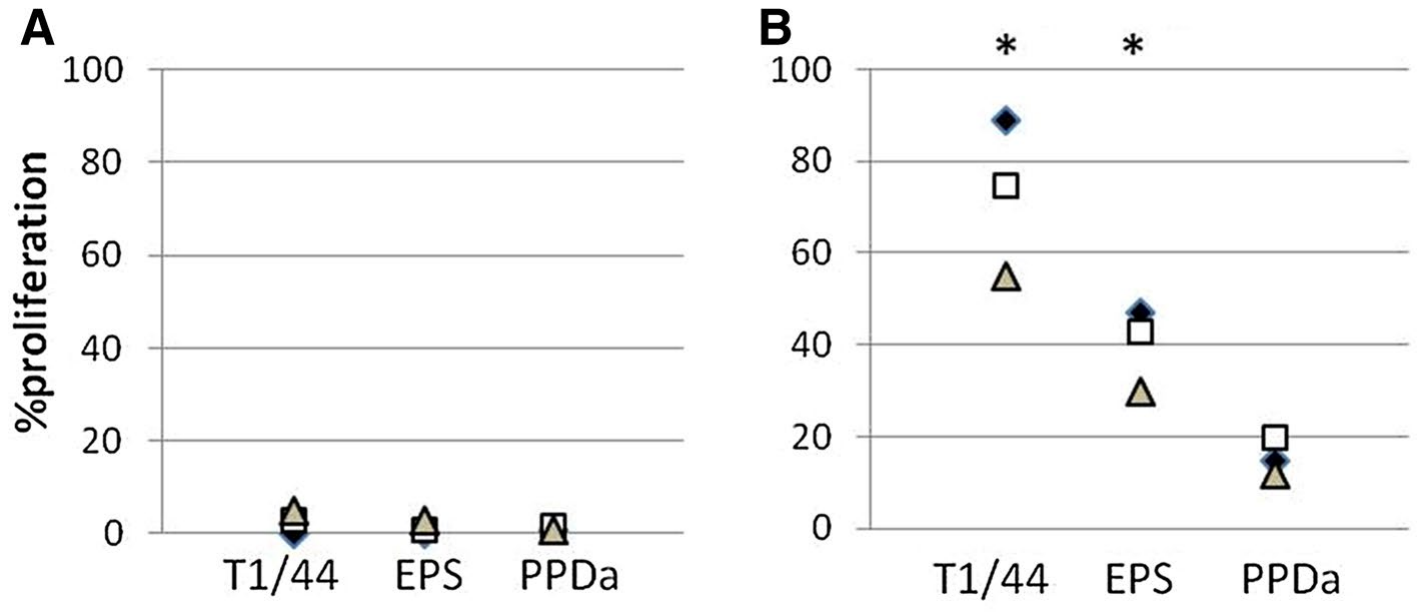
**Effect of free** ***Mmm*** **galactan on recall proliferation of CD4+ T lymphocytes from naïve cattle (** **A** **) and CBPP-infected cattle (** **B** **).** CFSE-labeled cells were stimulated for 7 days with whole inactivated *Mmm* (T1/44, 5 μg/mL), galactan (EPS, 10 μg/mL) or PPDa (10 μg/mL). Different symbols represent the net effect of stimulations (i.e., stimulated cultures minus non-stimulated cultures) for each animal individually (*n* = 3). Asterisks indicate statistically significant differences with PPDa.

As Th1 cells belong to the CD4+ lineage of T lymphocytes, we focused on that particular sub-population. No proliferation was detected upon in vitro stimulation of CFSE-labeled CD4+ T cells from naïve animals with *Mmm* galactan (Figure 5A). On the other hand, *Mmm*-experienced CD4+ T cells proliferated in response to both galactan and whole inactivated *Mmm* in a specific manner as demonstrated by a statistically significant higher percentage of proliferation in comparison to irrelevant PPDa antigen (Figure 5B). Finally, analysis of lymphocytes other than CD4 (i.e., the CD4− population comprising B cells, CD8+ and γδTCR+ T cells) revealed a significant proliferation but exclusively in cultures that were stimulated with whole inactivated *Mmm* (Additional file 2).

**Figure 6:**
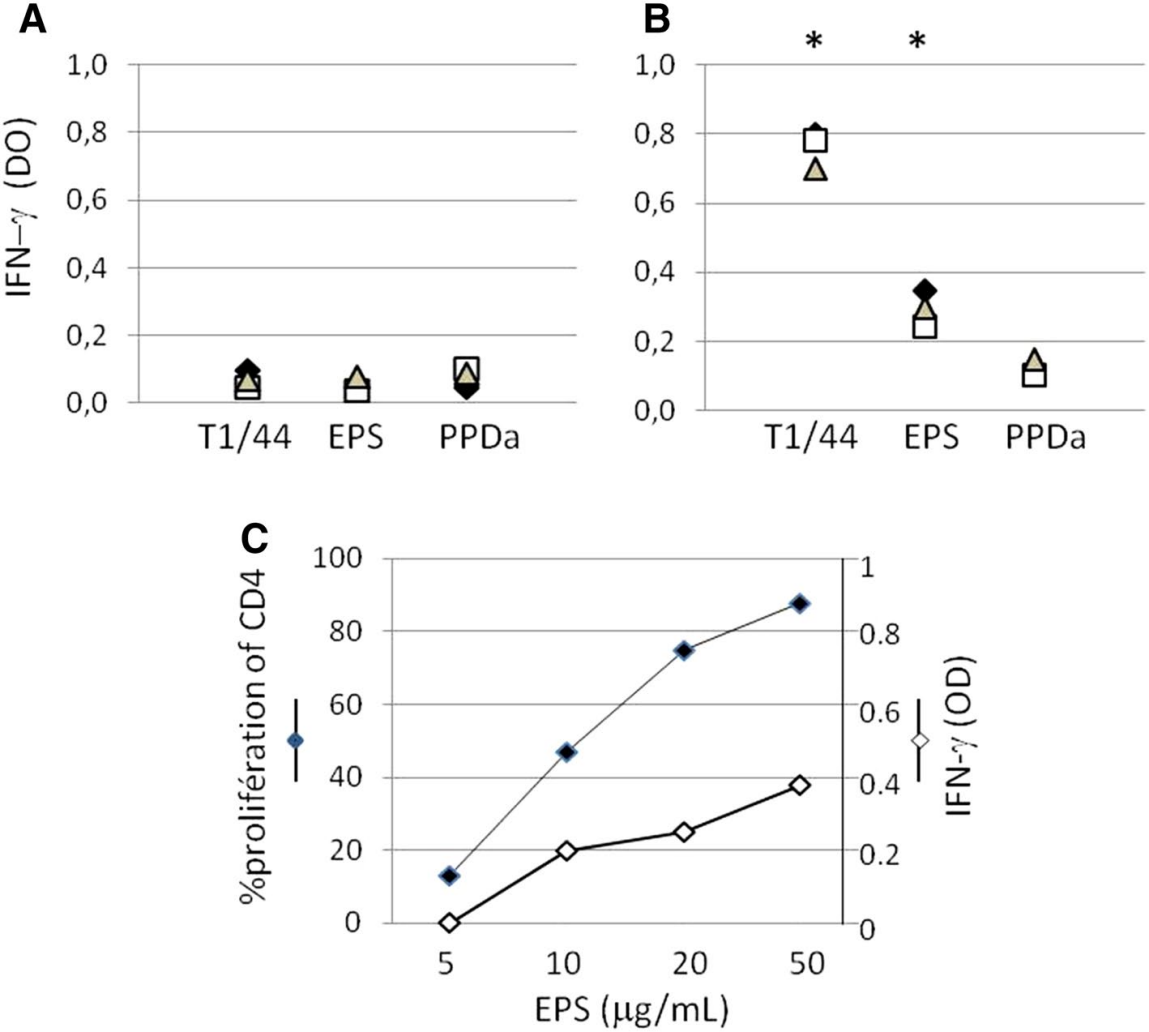
**Effect of free** ***Mmm*** **galactan on recall IFN-γ responses from naïve cattle (** **A** **) and CBPP-infected cattle (** **B** **).** Cells were stimulated for 7 days with whole inactivated *Mmm* (T1/44, 5 μg/mL), galactan (EPS, 10 μg/mL) or PPDa (10 μg/mL). Different symbols represent the net effect of stimulations (i.e., stimulated cultures minus non-stimulated cultures) for each animal individually (*n* = 3). Asterisks indicate statistically significant differences with PPDa. **C** Dose effect of galactan (EPS) on CD4 proliferation and IFN-γ responses.

The secretion of the Th1 cytokine IFN-γ was measured in cultures supernatants. As observed for proliferation, specific recall IFN-γ responses were detected only when cells from CBPP-infected cattle were used (Figures 6A and B). In these animals, galactan induced significant IFN-γ responses although at much lower levels than those obtained with inactivated *Mmm* (Figure 6B). Increasing the dose of galactan did not allow matching of IFN-γ responses obtained with inactivated *Mmm* despite comparable proliferation levels (Figure 6C).

## Discussion

The *Mmm* polysaccharide, galactan, is highly immunogenic as shown by antibody responses detected during infection and characterized by a very rapid and high level of IgM directed towards polysaccharidic *Mmm* components [12]. However, at the same time, *Mmm* that multiply in lung lesions are secreting free galactan EPS but there is no data available concerning the immunomodulatory properties of this compound. In this study, we investigated the impact of EPS on innate immunity through activation of bovine macrophages and naïve lymphocytes, and, on adaptive immunity, through expression of co-stimulatory molecules on macrophages and recall activation of *Mmm*-experienced lymphocytes.

The first step of this study was to identify a receptor for galactan among TLRs given their essential role in the early recognition of bacterial infections by the host immune system. Galactan was shown to interact with TLR2 which was supporting evidence for a potential involvement in innate immunity through interaction with TLR2-expressing cells such as macrophages and T lymphocytes [29]. The high purity grade of the galactan preparation used in our study was shown previously by silver staining on SDS-PAGE and nuclear magnetic resonance spectroscopy (NMR) [10]. However, as reported by Geurtsen et al. [35] for α-glucan purified from *Mycobacterium tuberculosis*, lipopeptides can be present at concentrations not detectable even by NMR and still induce substantial TLR2-dependent activity. In addition, the authors showed that these lipopeptides were successfully removed by phenol treatment [35]. In our model, we observed that reporter HEK cells could easily detect TLR2 activity triggered by FSL1, a lipopeptide produced by *Mycoplasma salivarium*, even at concentrations as low as 100 pg/mL (Additional file 3B). Therefore, we tested phenol treatment and showed that it did not significantly affect the TLR2 activity of galactan whereas it completely abrogated the activity of FSL1 (Additional file 3).

In contrast to TLR2-expressing HEK cells, we could not detect any activation of naïve lymphocytes by galactan as shown by the absence of cytokine release, proliferation, or even morphological changes (i.e., blastogenesis and cell death). Also, galactan had no measurable effects on naïve CD4+ T lymphocytes and this was not due to cytotoxicity. On the other hand, interaction between galactan and bovine macrophages resulted in a substantial release of IL-10, whereas low IL-12p40 and no TNF-α, both pro-inflammatory cytokines, were induced in these cells. In addition, the anti-inflammatory activity of galactan was confirmed by its ability to counteract the activity of LPS, a potent mediator of inflammation. Indeed, pre-treatment of macrophages by galactan dramatically reduced TNF-α and IL-12p40 responses triggered by LPS but also significantly increased (i.e., up to 300%) the production of IL-10. The lack of pro-inflammatory activity of certain polysaccharides and their capacity to inhibit LPS pro-inflammatory activity has been reported previously [30–34] and notably for another mycoplasmal EPS [30]. Moreover, α-glucan from *Mycobacterium tuberculosis* is known to amplify the LPS-mediated production of IL-10 by dendritic cells [35]. However, a dual effect, as described here for *Mmm* galactan and consisting in the concomitant inhibition of LPS pro-inflammatory activity and amplification of LPS anti-inflammatory activity via IL-10, has not been reported for other polysaccharides to the best of our knowledge. The galactan-mediated reduction of LPS-induced TNF-α release by macrophages may be explained by IL-10 acting on transcription factors such as STAT3 [36]. On the other hand, the amplification effect of galactan on LPS-induced IL-10 is probably more complex. For example, certain lipoglycans and glycans from *Mycobacterium tuberculosis* induce both prolonged and increased IL10 transcription in LPS-activated cells through interaction with C-type lectins rather than TLRs [35, 37]. Assessing the precise contribution of TLRs and C-type lectins in our model will await availability of reagents appropriate for use with bovine cells. Importantly, as far as CBPP is concerned, lipoproteins rather than LPS are in the top list of putative pro-inflammatory *Mmm* molecules [38]. Interestingly, IL-10 has also been shown to dampen the pro-inflammatory activity of lipoproteins [39, 40].

Galactan EPS from *Mmm* increased the expression of CD40 and CD80 co-stimulatory molecules on the surface of macrophages. In addition, galactan-specific recall proliferation of CD4+ T lymphocytes and IFN-γ responses were detected among lymphocytes draining the lungs of CBPP-infected cattle. However, cell mediated immune responses against galactan differed from recall responses triggered by whole inactivated *Mmm* in several ways. First, only CD4+ T lymphocytes proliferated in response to galactan whereas both CD4+ and CD4− T lymphocytes (most likely B lymphocytes as reported previously [25]) responded to *Mmm*. Second, both proliferation and IFN-γ recall responses induced by galactan were significantly lower (*p* < 0.05) than those triggered by *Mmm*. Third, a high amount of galactan was needed to match *Mmm*-induced proliferation of CD4+ T lymphocytes but IFN-γ levels remained substantially lower. Thus, the galactan-specific CD4+ T lymphocytes elicited in these animals are relatively poor producers of the Th1 cytokine when compared to their *Mmm*-specific homologues. A more complete cytokine profiling, including Th2, Th17 and Th22 cytokines, may be required in order to better characterize these responses.

In summary, we have shown here that galactan EPS, the free soluble form of *Mmm* polysaccharide, possesses anti-inflammatory properties and induces the production of IL-10 by macrophages. This result provides a likely explanation for the progressive increase in IL-10 detected in the plasma of cattle undergoing experimental CBPP [4]. Moreover, large amount of galactan are found in the blood of animals undergoing acute CBPP [12] which is also consistent with higher levels of plasma IL-10 detected in these animals when compared to animals with chronic CBPP [4]. In addition to multiple inhibitory effects on innate immunity (e.g., inhibition of pro-inflammatory cytokine release by macrophages [41, 42], and reduction of macrophage microbicidal activity [43]), IL-10 may also attenuate adaptative immunity by down-regulating the expression of IL-2 receptor on bovine T lymphocytes [44]. Thus, galactan may potentially also depress T cell responses directed at *Mmm* proteins. Interestingly, lower *Mmm*-induced recall activation of CD4+ T lymphocytes was detected in draining lymph nodes of animals chronically infected (i.e., presence of *Mmm* -containing sequestra in the lungs) in comparison to animals showing complete recovery [26]. Also, we have recently reported the absence of measurable T-cell responses in cattle one month after immunization with the attenuated T1/44 vaccine strain of *Mmm* [45]. The T1/44 strain also produces the EPS form of galactan [46] which may therefore potentially contribute to its poor T-cell immunogenicity.

All together our results suggest that free *Mmm* galactan, or EPS, is not involved in the inflammatory reaction which is the hallmark of acute CBPP. Thus, more work is needed to evaluate pro-inflammatory properties of other components of the *Mmm* membrane, including the CPS form of galactan and lipoproteins, as well as the *Mmm* secretome as a whole. In addition, our results suggest that EPS may dampen the host innate and possibly also acquired cell-mediated immune responses through the production of immunosuppressive IL-10. The role of other well-known mediators of innate immunity such as chemokines, but also other immunocompetent cells such as NK cells and dendritic cells deserves in-depth investigation.

## Additional files

10.1186/s13567-015-0252-6 **Effect of free** ***Mmm*** **galactan on surface expression of CD40 on macrophages.** Cells were incubated for 48 h with either galactan (EPS) at 0.1, 1 and 10 µg/mL, or with *E. coli* LPS and medium only (C-) as positive and negative controls respectively. The geometric mean fluorescence intensity (MFI) of the CD40 marker was measured by flow cytometry and results expressed as mean (±SD) values of three different experiments. Asterisks indicate statistically significant differences (*p* < 0.05) with the negative control.
10.1186/s13567-015-0252-6 **Analysis of in vitro recall proliferation among non CD4 (CD4−) bovine lymphocytes.** Recall proliferation of CFSE-labeled CD4**−** lymphocytes from naïve (A) and CBPP-infected cattle (B) after stimulation with whole inactivated *Mmm* (T1/44, 5 μg/mL), free *Mmm* galactan (EPS, 10 μg/mL) or PPDa (10 μg/mL). Different symbols represent the net effect of stimulations (i.e., stimulated cultures minus non-stimulated cultures) for each animal individually (*n* = 3). Asterisks indicate statistically significant differences with PPDa.
10.1186/s13567-015-0252-6 **Effect of phenol extraction on TLR2-galactan and TLR2-FSL1 interactions.** Phenol extraction has a negligible effect on free *Mmm* galactan-induced secretion of embryonic alkaline phosphatase (SEAP) release from HEK-Blue mTLR2 cells (tlr2) (A). In contrast, TLR2-driven SEAP activity of the *Mycoplasma salivarium* lipopeptide FSL1 is almost completely inhibited after phenol extraction (B). Galactan (EPS) was extensively dialysed after phenol extraction and used at 2 μg/mL final concentration. FSL1 could not be dialysed due to its low molecular weight (i.e., 1.6 kDa) but it was used at a final concentration of 100 pg/mL which allowed sufficient dilution of residual phenol to avoid cytotoxicity. SEAP release is expressed as mean fold changes (±SD) in comparison to unstimulated cells (*n* = 3).
