# Supplementary figures and images for: Free exopolysaccharide from Mycoplasma mycoides subsp. mycoides possesses anti-inflammatory properties
Source: Vet Res. 2015 Oct 21;46:122. doi: 10.1186/s13567-015-0252-6 (PMC4618858; doi:10.1186/s13567-015-0252-6)

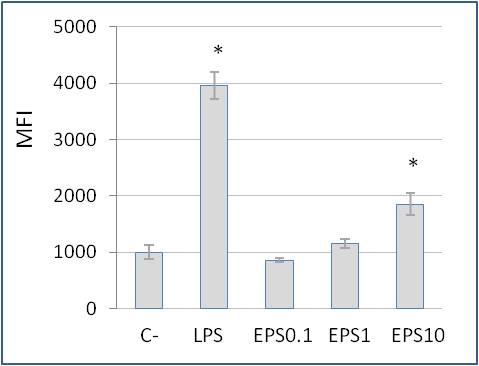

Supplement: Supplementary file 1 — 10.1186/s13567-015-0252-6 Effect of free Mmm galactan on surface expression of CD40 on macrophages. Cells were incubated for 48 h with either galactan (EPS) at 0.1, 1 and 10 µg/mL, or with E. coli LPS and medium only (C-) as positive and negative controls respectively. The geometric mean fluorescence intensity (MFI) of the CD40 marker was measured by flow cytometry and results expressed as mean (±SD) values of three different experiments. Asterisks indicate statistically significant differences (p < 0.05) with the negative control. [file 13567_2015_252_MOESM1_ESM.tif]

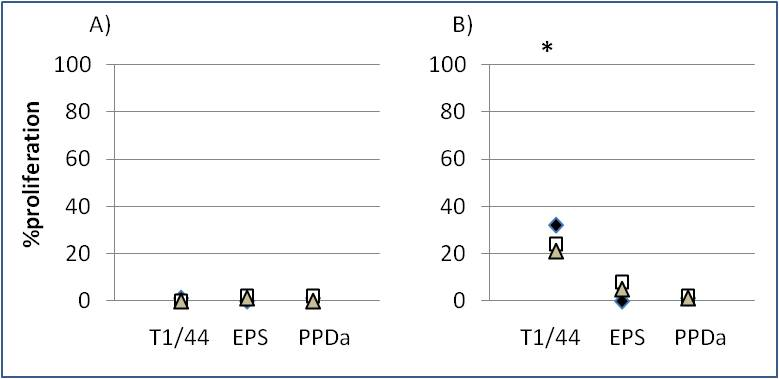

Supplement: Supplementary file 2 — 10.1186/s13567-015-0252-6 Analysis of in vitro recall proliferation among non CD4 (CD4−) bovine lymphocytes. Recall proliferation of CFSE-labeled CD4− lymphocytes from naïve (A) and CBPP-infected cattle (B) after stimulation with whole inactivated Mmm (T1/44, 5 μg/mL), free Mmm galactan (EPS, 10 μg/mL) or PPDa (10 μg/mL). Different symbols represent the net effect of stimulations (i.e., stimulated cultures minus non-stimulated cultures) for each animal individually (n = 3). Asterisks indicate statistically significant differences with PPDa. [file 13567_2015_252_MOESM2_ESM.tif]

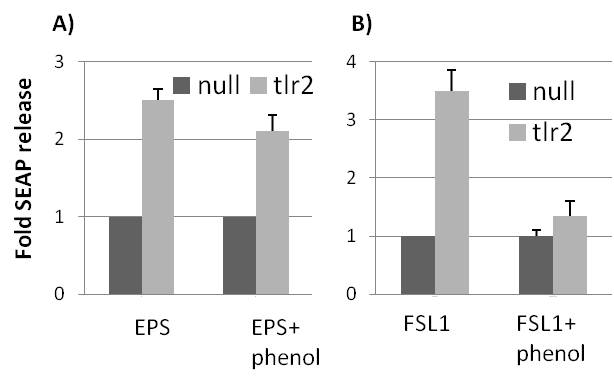

Supplement: Supplementary file 3 — 10.1186/s13567-015-0252-6 Effect of phenol extraction on TLR2-galactan and TLR2-FSL1 interactions. Phenol extraction has a negligible effect on free Mmm galactan-induced secretion of embryonic alkaline phosphatase (SEAP) release from HEK-Blue mTLR2 cells (tlr2) (A). In contrast, TLR2-driven SEAP activity of the Mycoplasma salivarium lipopeptide FSL1 is almost completely inhibited after phenol extraction (B). Galactan (EPS) was extensively dialysed after phenol extraction and used at 2 μg/mL final concentration. FSL1 could not be dialysed due to its low molecular weight (i.e., 1.6 kDa) but it was used at a final concentration of 100 pg/mL which allowed sufficient dilution of residual phenol to avoid cytotoxicity. SEAP release is expressed as mean fold changes (±SD) in comparison to unstimulated cells (n = 3). [file 13567_2015_252_MOESM3_ESM.tif]
